# Effect of Storage and Drying Treatments on Antioxidant Activity and Phenolic Composition of Lemon and Clementine Peel Extracts

**DOI:** 10.3390/molecules28041624

**Published:** 2023-02-08

**Authors:** Esther Gómez-Mejía, Iván Sacristán, Noelia Rosales-Conrado, María Eugenia León-González, Yolanda Madrid

**Affiliations:** Department of Analytical Chemistry, Faculty of Chemistry, Complutense University of Madrid, 28040 Madrid, Spain

**Keywords:** polyphenols, antioxidant activity, citrus peels, liquid chromatography, drying treatments, storage conditions, multivariate analysis

## Abstract

Obtaining polyphenols from horticultural waste is an emerging trend that enables the valorization of resources and the recovery of value-added compounds. However, a pivotal point in the exploitation of these natural extracts is the assessment of their chemical stability. Hence, this study evaluates the effect of temperature storage (20 and −20 °C) and drying methods on the phenolic composition and antioxidant activity of clementine and lemon peel extracts, applying HPLC-DAD-MS, spectrophotometric methods, and chemometric tools. Vacuum-drying treatment at 60 °C proved to be rather suitable for retaining the highest antioxidant activity and the hesperidin, ferulic, and coumaric contents in clementine peel extracts. Lemon extracts showed an increase in phenolic acids after oven-drying at 40 °C, while hesperidin and rutin were sustained better at 60 °C. Hydroethanolic extracts stored for 90 days preserved antioxidant activity and showed an increase in the total phenolic and flavonoid contents in lemon peels, unlike in clementine peels. Additionally, more than 50% of the initial concentration was maintained up to 51 days, highlighting a half-life time of 71 days for hesperidin in lemon peels. Temperature was not significant in the preservation of the polyphenols evaluated, except for in rutin and gallic acid, thus, the extracts could be kept at 20 °C.

## 1. Introduction

Bioactive compounds, derived from plants and other natural sources, have attracted increasing attention in view of their key role in maintaining, promoting, and prolonging quality and productive life [1]. In addition, for some years now, the replacement of chemically synthesized additives (e.g., the antioxidant butylated hydroxytoluene, commonly known as BTH) by natural functional and nutritional ingredients, which ensure greater safety and health benefits for consumers, has been increasingly sought after [2,3]. In this regard, polyphenols are a group of secondary metabolites of natural origin present as essential physiological compounds in plants and plant-based products, such as fruits, vegetables, cereals, and beverages, among others [4]. Currently, they have acquired great interest in the food, pharmaceutical, and cosmetic industries given their numerous bioactive properties, emphasizing their antioxidant, anti-inflammatory, antiproliferative, anti-allergic, antiviral, anticarcinogenic, neuroprotective, and antimicrobial properties [5].

Obtaining polyphenols from waste and natural by-products generated by the food industry is an emerging trend that enables the valorization and reuse of resources as raw materials in other industries. Moreover, it contributes as much to the minimization of environmental constrains triggered by food waste disposal (e.g., landfill occupation, greenhouse gas emissions, and resource expenditure) as to the attainment of economic and social benefits, thereby supporting the achievement of Sustainable Development Goal 12: “Responsible consumption and production” [6,7]. Within the agri-food sector, one of the most prominent industries is the citrus juice and citrus-based food products industry, with the annual production of more than 90 million tons in 2022 [8], of which approximately 50–60% of the total fruit weight was discarded as waste in the form of peel, seeds, and membranes. Generally, the peels are rather plentiful in polyphenols as they protect the fruits from external hazards, and thus, significant interest has been shown in recycling them as a source of natural bioactive polyphenols [9]. Namely, catechin, rutin, ferulic acid, and *p*-coumaric acid have been recovered from orange (*Citrus sinensis*) peels [10], while chlorogenic acid, naringenin, and phloretin were found in mandarin (*Citrus reticulata* Blanco) peels at levels between 18 and 1.8 mg·g^−1^ [11]. In addition, clementine and lemon peels were described to be particularly abundant in hesperidin and naringin, with concentrations up to 600 mg·g^−1^ [12]. Hence, citrus peels represent a promising source of natural phenolic extracts, with potential applications in the development of skin care products, natural dyes, functional foods, active packaging, and nutraceuticals [5,13]. In this context, the industrial production of bioactive extracts from horticultural by-products usually generates liquid extracts. Nonetheless, sometimes a total or partial drying process is often applied to the extracts to reduce their volume and/or improve their handling and application properties [14]. Cold storage, thermal treatments (e.g., hot air drying), or freeze-drying are some of the most common processes by which agri-food matrices and their natural extracts are subjected for their preservation in liquid and solid states [14,15]. However, a pivotal point in the further exploitation of these bioactive extracts is the evaluation of their chemical stability after processing and storage, since it is well established that polyphenols are rather sensitive to physical processes, temperature, pH, light, and oxygen availability. Therefore, changes in these factors can promote modifications in the native composition, antioxidant activity, and the bioavailability of the phenolic extracts [15]. So far, few studies have dealt with the stability of polyphenols in horticultural residues after drying and storage processes, let alone that of the final phenolic extracts. Specifically, Li et al., investigated the impact of temperature (50–80 °C) on the antioxidant activity and phenolic content of mandarin peels dried by hot air, observing a 29–60% decrease in the total content of polyphenols (TPC) and flavonoids, as well as a decrease in their ability to scavenge DPPH radicals [16]. Likewise, Papoutsis et al. studied the effects of freeze-dying, hot air-drying, and vacuum-drying on the phenolic composition and antioxidant activity of lemon pomace, highlighting that the freeze-dried samples retained less rutin, coumaric, and gallic acids, in addition to exhibiting lower antioxidant activity [17]. On the other hand, Castillo et al., analyzed the stability of a grape marc phenolic extract in 50% ethyl lactate when stored under different conditions of temperature (4, 20 and −20 °C), light (presence and absence), and oxidative exposure for 62 days, observing that the DPPH scavenging capacity and the TPC did not fluctuate significantly upon changes in light and temperature. In addition, a 200% increase in the concentration of gallic acid and quercetin, and a decrease of up to 63% in the concentration of rutin, were reported [14]. Hence, it appears that the behavior of the phenolic profile and the antioxidant activity of extracts are quite heterogeneous and depend on the matrix, the phenolic nature, and the operating conditions under which they are preserved, emphasizing the need to know and control both the appropriate preservation conditions and the most suitable drying treatments to ensure the quality of the final product.

In view of the above, this work performs an exhaustive analysis of the effect of storage temperature (20 and −20 °C) and different drying techniques operating at 40 and 60 °C, including oven and vacuum drying, on the phenolic composition and antioxidant activity of hydroethanolic extracts obtained from clementine and lemon peels.

## 2. Results and Discussion

### 2.1. Performance of the cLC-DAD-MS Method

This study developed, optimized, and validated a cLC-DAD-MS analytical method for the determination of myricetin, resveratrol, quercetin, kaempferol, rutin, and hesperidin, as well as for that of gallic, dihydroxybenzoic, caffeic, *p*-coumaric, and *trans*-ferulic acid. Due to its superior sensitivity, DAD was used to quantify myricetin, resveratrol, rutin, and hesperidin. Furthermore, due to the low sensitivity frequently associated with the negative ionization mode [18,19,20,21,22,23] and the inability of hesperidin and rutin to be ionized under optimum chromatographic conditions, neither substance was detected by the MS analyzer. However, the sensitivity of MS was superior for gallic, DHB, caffeic, and *p*-coumaric acids, and for quercetin and kaempferol. Lastly, the sensitivity of *trans*-ferulic acid was comparable for both DAD and MS, although measurement by MS was favored due to its greater detection selectivity. Table 1 contains mass spectral data, calibration curves, and wavelengths for the identification and quantification of polyphenols by cLC-DAD-MS.

On the other hand, the analytical parameters of the cLC-DAD-MS method are shown in Table 2, where wide linear ranges are found, namely for gallic and dihydroxybenzoic acids (20–450 μg·L^−1^). Further, good linearity was also noted for all the analytes studied at concentrations within the ranges tested, with coefficients of determination (R^2^) between 0.9900 (caffeic acid) and 0.9993 (hesperidin). Acceptable and diverse LODs between 0.1 and 20 μg-L^−1^, and LOQs between 0.3 and 67 μg-L^−1^, were also estimated for quercetin and kaempferol, and for rutin and myricetin, respectively. Moreover, repeatability and intermediate precision were assessed for each polyphenol, as described in Section 3.8. The intra-day precision ranged between 0.32 (resveratrol and quercetin) and 8.6% (dihydroxybenzoic acid) for the retention factor, and between 1.0 (hesperidin) and 6.5% (quercetin) for the peak areas. Regarding inter-day variations, the maximum RSD values found were 8.0% (dihydroxybenzoic acid) for the retention factor and 5.9% (*p*-coumaric acid) for the peak area. Therefore, a satisfactory performance and the reproducibility of the method was achieved for all the analytes studied (RSD < 10%).

### 2.2. Chemical Characterization of the Phenolic Extracts from Citrus Peels

The extracts obtained, following the SLE procedures described in Section 3.3., were initially screened in terms of individual polyphenols, total phenolic content, and antioxidant activity (Table 3). As for the determination of the phenolic profile by cLC-DAD-MS, gallic, dihydroxybenzoic, caffeic, *p*-coumaric, and *trans*-ferulic acids, and rutin, hesperidin, myricetin, resveratrol, quercetin, and kaempferol were identified. However, the dihydroxybenzoic and caffeic acids myricetin, resveratrol, and quercetin were determined at the detection limit levels of the method and, therefore, were not quantifiable.

Overall, both extracts showed considerable values of total polyphenols—these being 10.4 and 16 mg·g^−1^ for lemon and clementine peels, respectively—which were significantly higher in clementine peels, demonstrating their potential as a source of bioactive polyphenols. Likewise, a higher abundance of the flavonoid family was observed in both extracts (Table 3), mainly due to the richness of hesperidin (8.7 mg·g^−1^ in lemon peels and 15 mg·g^−1^ in clementine peels) and rutin (1.33 ± 0.06 mg·g^−1^) in lemon peel extract. Conversely, the flavonol kaempferol was a minor compound detected at a concentration below 10 µg·g^−1^ in the samples studied (Table 3). As for phenolic acids, *trans*-ferulic acid was by far the richest in clementine peels (438 µg·g^−1^), together with *p*-coumaric acid for lemon peels (around 177 µg·g^−1^ for both phenolics), ultimately followed by gallic acid, whose concentration was significantly lower in lemon peels (Table 3). Consistent with the findings presented, hesperidin has also been described as one of the major polyphenols in lemon and clementine peels, exhibiting significant protective activity against ultraviolet and ionizing radiation [16,24,25] as well as a greater amount of *trans*-ferulic and *p*-coumaric acid in clementine peels compared to lemon peels [12].

Moreover, raw citrus extracts were also characterized by spectrophotometric methods. As can be seen in Table 3, the values of TPC and TFC were significantly higher in clementine peel extracts (6.2 ± 0.8 mg GAE·g^−1^ and 40 ± 2 mg QE·g^−1^, respectively), which is in agreement with the previously reported chromatographic results. Meanwhile, raw lemon peel extracts showed significantly higher antioxidant activity (IC_50_ = 0.81 mg·g^−1^), which may be attributed to the limited ability of hesperidin to inhibit DPPH radicals [12] as a compound whose concentration is higher in clementine peels.

### 2.3. Stability of Polyphenols under Drying Treatments

#### 2.3.1. Effect of Drying Treatment on the Spectrophotometric Data

**Figure 1:**
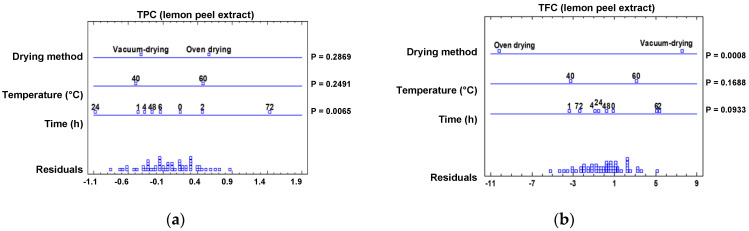

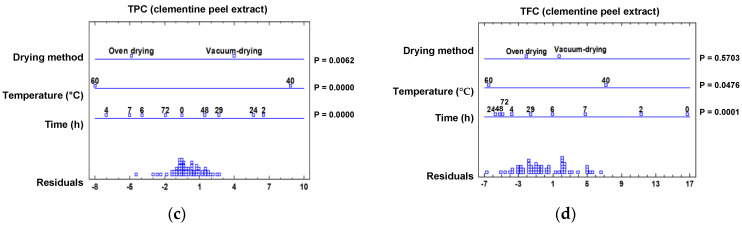
Multifactorial ANOVA plots showing the influence of time, temperature, and extract drying method on: (**a**) TPC in lemon peel extracts, (**b**) TFC in lemon peel extracts, (**c**) TPC in clementine peel extracts, and (**d**) TFC in clementine peel extracts. *p*-values (P) lower than 0.05 indicate a statistically significant effect at 95% confidence level.

In this study, the TPC and TFC of the phenolic extracts of clementine and lemon peel subjected to hot air and vacuum-drying treatments at 40 °C and 60 °C were determined at different time intervals until the total dryness was reached (Table S1), these intervals being 72 h for the oven-drying of both peels at 40 °C, 29 h for the oven-drying of clementine peels at 60 °C, 24 h for the oven-drying of lemon peels at 60 °C, 7 h for the vacuum-drying of clementine peels at 40 °C, 6 h for the vacuum-drying of clementine peels at 60 °C as well as for the vacuum-drying lemon peels at 40 °C, and 4 h for the vacuum-drying of lemon peels at 60 °C. So, to identify the influence of the drying treatment, temperature, and time, a multifactorial analysis of variance (ANOVA) was carried out, as shown in Figure 1.

The results of this statistical analysis revealed a significant variation under the different drying factors, with rather diverse behavior of both the TPC and TFC in the extracts analyzed. As for the drying method, a significant impact on the TFC of lemon peel extract and on the TPC of clementine extract was observed (Figure 1b,c), the values of which were higher than when vacuum-drying was performed. This difference could be ascribed to the lower oxidation degree of some phenolic compounds when extracts are dried in the absence of oxygen [17]. Additionally, temperature was decisive in the drying of the clementine peel extract (Figure 1c,d), showing a significant improvement of both parameters at 40 °C, attributable to the greater degradation of some thermolabile phenolic compounds at higher temperatures [15]. Then again, both measurements were time-dependent, other than for the TFC in the lemon peel extract (*p*-value > 0.05). It is worth noting the increases (*p*-value < 0.05) observed in the TPC with respect to the initial extract, which were up to 35% after 24 h of drying the clementine extracts and 24% after 72 h of drying the lemon extracts. In this sense, previous studies on the stability of phenolic compounds have shown similar patterns of behavior, revealing unique increases due to the degradation of larger polymeric or glycosylated phenolic chains. In addition, the reactivity of these simpler phenolic compounds could be higher with respect to the Folin–Ciocalteu method, leading to higher rates [14,17].

**Figure 2:**
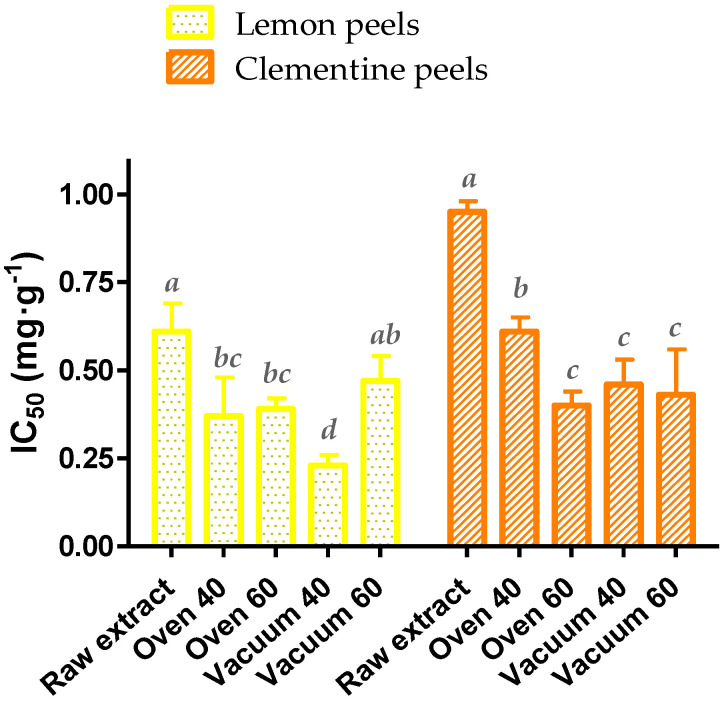
Antioxidant capacity based on DPPH assay of raw and dried extracts with different treatments of lemon and clementine peels. Data with different superscripts are significantly different at *p*-value < 0.05, according to one-way ANOVA and Fisher’s LSD test.

At the same time, the antioxidant activity of the dried phenolic extracts of citrus peels was determined according to the DPPH scavenging method. A comparison of the IC_50_ values of dried extracts versus those of the initial ones (raw extract) will allow an estimation of the quality and the potential use of the dried extracts. Figure 2 shows the antioxidant results, revealing a significant improvement of the radical inhibition ability after all drying treatments other than for the vacuum-dried lemon peel extract at 60 °C (*p*-value > 0.05).

Concerning the oven-dried clementine peel extracts, a decrease in IC_50_ was found with rising temperatures, as higher temperatures may cause the release of some phenolic acid and flavonoid complex chains, as well as reduce the activity of polyphenol oxidase [17]. However, this pattern was not reported in vacuum-dried peel extracts, since the time required to reach total dryness was considerably lower (<7 h). Similarly, Xu et al. reported an increase in the total antioxidant activity of oven-dried grapefruit peels when the temperature was increased from 90 °C to 120 °C [26]. Inversely, no difference was observed for lemon peel extracts in both oven-dried samples, while a 62% gain in DPPH scavenging activity (*p*-value < 0.05) was found for the extract dried under vacuum at 40 °C, indicating that this may be the most suitable method for drying the latter. Moreover, this antioxidant enhancement could be attributed to the release of some flavonoids at lower temperatures, as previously described in vacuum-drying lemon pomace [17]. Overall, the different patterns of antioxidant activity and the total phenolic contents revealed in both extracts could be ascribed not only to the diverse polyphenol composition, but also to the presence of other natural compounds, emphasizing the key role of the matrix and the extraction medium in studying the stability of natural extracts.

#### 2.3.2. Effect of Drying Treatment on the Individual Phenolic Composition

Despite the fact that spectrophotometric methods have been extensively applied for estimating either overall phenolic content or antioxidant activity, their limited selectivity can lead to misleading results [27]. Hence, to thoroughly understand the effect of the drying treatment on the stability of the phenolic extracts from citrus peels, those approaches should be combined with the determination of the individual phenolic composition. Thus, the concentrations of hesperidin, gallic, *p*-coumaric, and *trans*-ferulic acid were monitored under every drying condition in both citrus extracts. However, kaempferol was not included in the study, given that it could not be quantified once the drying process was initiated, and neither was the gallic acid in the lemon peel extracts.

**Figure 3:**
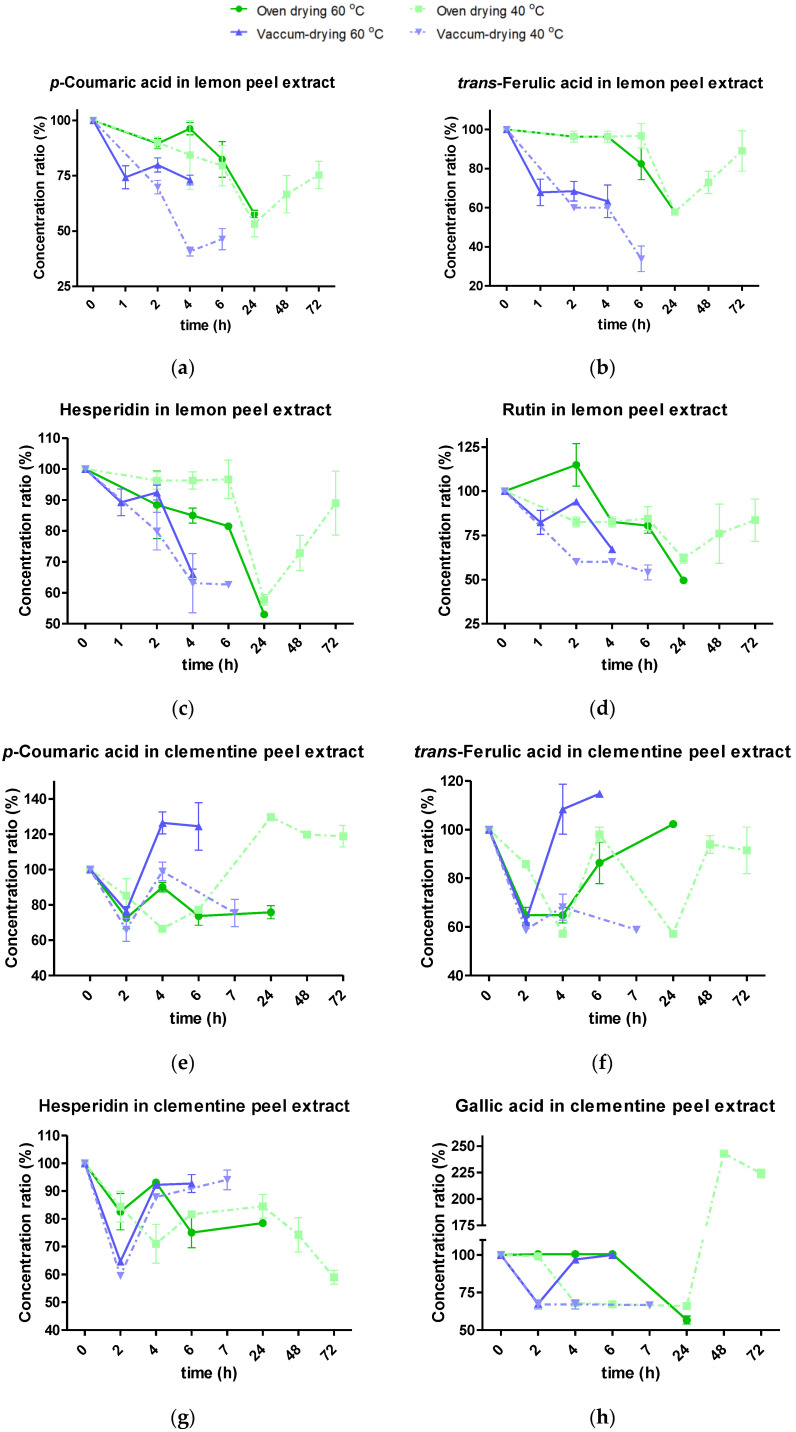
Stability of the individual polyphenols monitored in lemon peel extract (**a**–**d**) and clementine peel extract (**e**–**h**) under different drying treatments.

The evolution of the aforementioned phenolic compounds characterized in the citrus extracts under each thermal treatment is summarized in Figure 3, where the results are expressed as percentage ratios in relation to the initial concentration (i.e., the percentage of the concentration determined for each analyte at the drying time, with respect to the initial one), to facilitate the visualization of the phenolic changes.

In relation to the drying processes of citrus peel extracts, it is widely-accepted that heat treatments tend to yield a drop in phenolic content [11]. Consistent with this statement, significant losses were observed in all the studied polyphenols of the completely dried lemon extracts (final concentration rate between 33% and 73%), apart from the oven-dried lemon extract at 40 °C (*p*-value > 0.05) (Figure 3a–d). On the other hand, the behaviors of the polyphenols of the dried clementine peels were more dispersed. Hesperidin, gallic, and *trans*-ferulic acids preserved less than 59% of the initial concentration after undergoing oven-drying at 40 °C, oven-drying at 60 °C, and vacuum-drying at 40 °C, respectively, whereas *p*-coumaric acid was particularly resilient, with a minimum concentration rate of 75% after vacuum-drying at 40 °C. Comparably, the reduction of chlorogenic acid, hesperidin, and naringenin contents during the oven-drying process of the clementine peels were confirmed, as well as a loss in rutin and neohesperidin content being confirmed when drying lemon pomace at increasing temperatures [11,17]. This phenolic degradation could be induced by the thermal cracking and the oxidation of the native phenolic forms, given the simultaneous heat and mass transfer caused by the heat flowing through the extract and the oxidizing action of polyphenol oxidase and oxygen, mainly in oven-drying [11]. Nevertheless, some significant rises were detected in the totally or partially dried extract (Figure 3). Here, a release of all phenolic acids and flavonoids studied was observed after 24 h of oven-drying treatments at 40 °C in both peel extracts, apart from hesperidin in clementine peel. In addition, a similar increase was observed after 4 h of vacuum drying at 60 °C in the clementine peel extract. Other authors have observed such changes in the final periods of drying treatments [11,28], attributing them to the depolymerization or dissociation of other phenolic chains into free polyphenols, especially when it comes to phenolic acids.

Thus, as can be seen in Figure 3e,f,h, the concentration ratios of gallic (224 ± 6, %), coumaric (114 ± 2, %), and ferulic (124 ± 19, %) acids were higher than the initial ones in the clementine extracts dried in the oven at 40 °C and those in the vacuum evaporator at 60 °C, respectively.

#### 2.3.3. Multifactorial Study of the Drying Treatments by Principal Component Analysis

**Figure 4:**
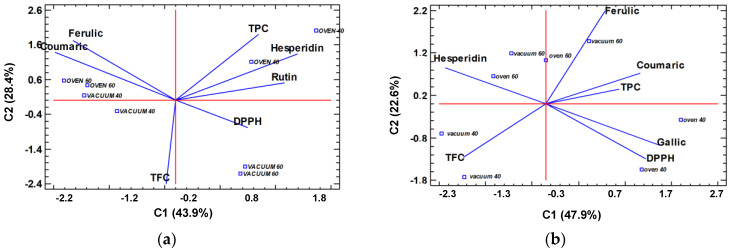
Principal component analysis biplot of the simultaneous evaluation of the loadings (individual phenolic concentration, TPC, TFC and DPPH) and scores (drying treatments) studied in: (**a**) lemon peel extracts and (**b**) clementine peel extracts.

To easily elucidate which drying conditions are more appropriate for maintaining the integrity and quality of clementine and lemon extracts, the spectrophotometric (TPC, TFC, and DPPH) and chromatographic (individual polyphenol content) data obtained after each of the drying treatments were simultaneously studied by principal component analysis (PCA). It is worth mentioning that each sample was analyzed independently due to the role of the matrix in the stability and the antioxidant activity of the final products [14]. As shown in Figure 4, both data sets were plotted to produce two-dimensional plots that reflected more than 70% of the total data variance.

Concerning lemon peel extracts (Figure 4a), it is possible to verify that the *trans*-ferulic acid and *p*-coumaric acid contents, along with their antioxidant capacity (lower DPPH value), were higher when the extract was oven-dried at 60 °C or vacuum-dried at 40 °C. Meanwhile, the stability of hesperidin and rutin, together with the TPC, were favored in oven-dried extracts at 40 °C. By contrast, vacuum-drying at 60 °C seemed to be the least favorable method in obtaining functional dried lemon peel extracts. On the other hand, thermal treatments at 60 °C, either in an oven or under vacuum, were quite effective in promoting the properties of clementine peel extracts. Thus, they favored the abundance of hesperidin, *trans*-ferulic acid, *p*-coumaric acid, TPC, and antioxidant activity. Finally, oven drying at 40 °C particularly boosted the concentration of gallic acid, mainly due to the degradation of bound phenolic chains after long drying times [28].

### 2.4. Stability of Polyphenols under Storage Conditions

#### 2.4.1. Effect of Storage Temperature on the Spectrophotometric Data

Extracts obtained from natural sources, including horticultural biowaste, using GRAS solvents such as ethanol, have been demonstrated to exert synergistic activity both in the overall extraction of phenolic compounds and in the inherent bioactivity [14]. Therefore, studying the stability of the natural liquid extract at different storage temperatures is quite convenient not only for determining the most appropriate storage condition, but also to understand how the extract behaves in the final product. In this regard, the TPC, TFC, and DPPH antioxidant activity of the hydroethanolic extracts of lemon and clementine citrus fruits, stored at 20 °C and −20 °C for 90 days, were monitored at different intervals (Table S2).

**Figure 5:**
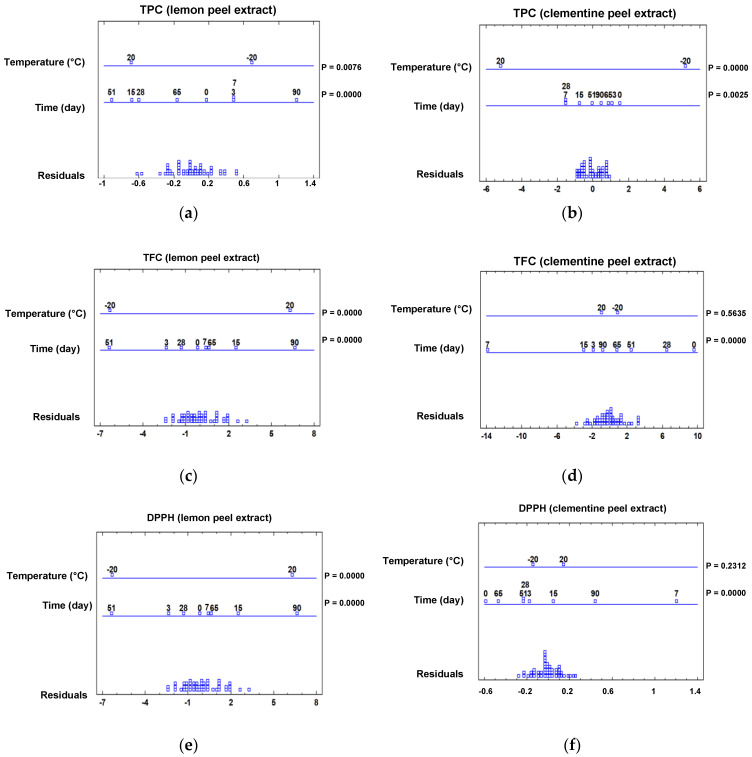
Multifactorial ANOVA plots showing the influence of storage time and temperature on: (**a**) TPC in lemon peel extracts, (**b**) TPC in clementine peel extracts, (**c**) TFC in lemon peel extracts, (**d**) TFC in clementine peel extracts, (**e**) DPPH in lemon peel extracts, and (**f**) DPPH in clementine peel extracts. *p*-values (P) lower than 0.05 indicate a statistically significant effect at 95% confidence level.

Figure 5 shows the effects of storage time and temperature on the main bioactive indices of the phenolic extracts from the lemon peels (Figure 5a,c,d) and clementine peels (Figure 5b,e,f). Statistical differences were found for each storage factor in every index of the lemon peel extract, while in clementine peel ones, temperature was only crucial when it came to the TPC, highlighting superior results when the extract was preserved at −20 °C. Regarding the behavior of phenolic indices over the storage time, an increase in the TPC and TFC values of lemon peel extracts was observed at 90 days of testing (*p*-value < 0.05), which again can be attributed to the release of free and more reactive polyphenols [14] and to the lower degradation of native polyphenols at −20 °C [29]. However, this increase did not correlate with an improvement in antioxidant activity, since the IC_50_ value was comparable at 0 and 90 days (*p*-value > 0.05). At the other end of the spectrum, clementine peel extract did not decrease its TPC at 90 days (Figure 5b), in contrast to the TFC and the antioxidative activity (Figure 5d,f). Similarly, Castillo et al. reported no significant effects of storage temperature (20, 4 and −20 °C) on the stability of the TPC of grape pomace extract, unlike on the DPPH antioxidant capacity, which decreased significantly after 62 days [14].

#### 2.4.2. Effect of Storage Temperature on the Individual Phenolic Composition

**Figure 6:**
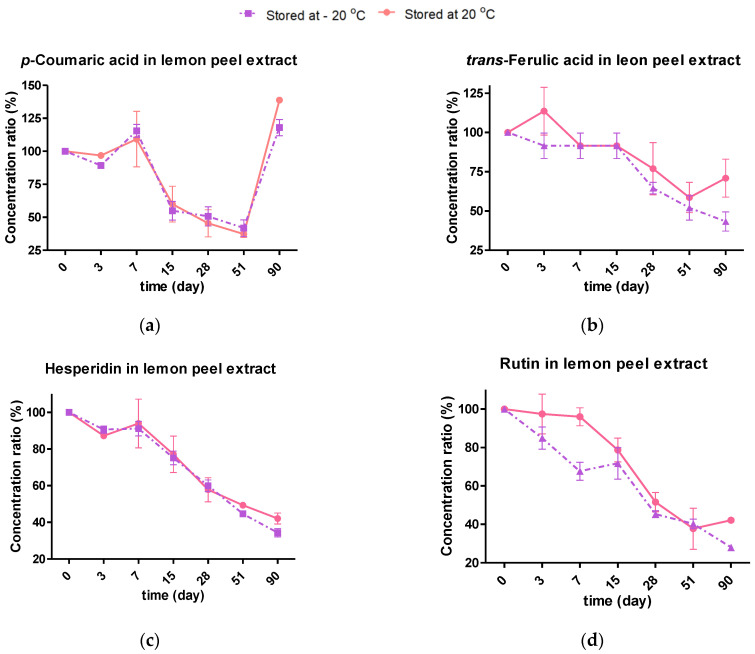

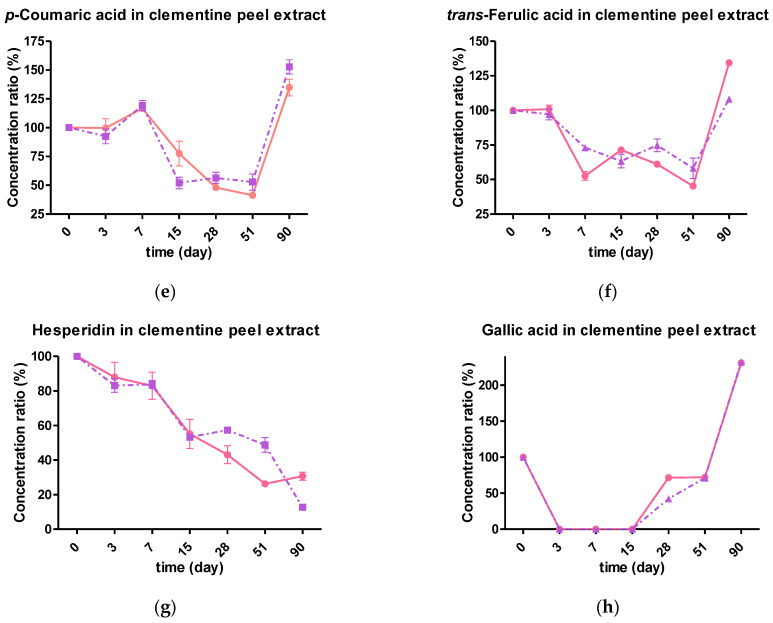
Stability of the individual polyphenols monitored in lemon peel extract (**a**–**d**) and clementine peel extract (**e**–**h**) during storage at 20 °C and −20 °C.

In a similar way to the drying study, the concentrations of hesperidin, gallic acid, *p*-coumaric acid, and *trans*-ferulic acid were examined at the two storage temperatures for 90 days. In general, a significant fluctuation in the profiles was observed, where there was a relationship between degradation and the formation of smaller polyphenolic compounds from others with larger structures, keeping them stable after 90 days in the case of the TPC in the clementine extract and the DPPH in the lemon extract. The statistical significance of the storage factors considered was determined by multifactorial ANOVA, disclosing that all polyphenols were significantly affected by storage time. On the other hand, the *p*-coumaric acid, *trans*-ferulic acid, and hesperidin concentrations were not temperature-dependent (*p*-value > 0.05) in contrast to rutin and gallic acid, whose stability was greater at 20 °C. In a similar way to that in this study, caffeic hydroxycinnamic acid showed no significant differences when stored at −20, 4 and 20 °C, whereas rutin and gallic acid were temperature-dependent [14]. Coumaric and ferulic acids proved to be quite stable in both extracts, maintaining at more than 50% for up to 51 days (Figure 6a,b,e,f). Furthermore, at 90 days, an increase in these acids was observed, reaching concentration ratios of up to 139%, apart from the ferulic acid present in the lemon extract (Figure 6a,b,e,f). Gallic acid, whose initial concentration in clementine extract was low (Table 3), was completely degraded after three days. However, after 28 days, it was detected again (concentration ratio of up to 72%) and followed an upward trend until 90 days, with a ratio of 232% (Figure 6h). Unlike the phenolic acids presented in this study, the larger flavonoids, such as rutin and hesperidin, showed rapid degradation with a continuously decreasing trend (Figure 6c,d,g). Indeed, less than 45% of the initial concentration was maintained after 90 days of testing. This pattern of behaviors was paralleled, as observed by Castillo et al. when studying the stability of grape pomace extracts. Indeed, a low stability of rutin, which degraded by 63% after 36 days of storage, as well as the increase in simple acids after long storage times (62 days) was reported. The latter can be attributed to the decomposition of larger structures containing phenolic monomers, such as hydrolysable tannins consisting of glucose esters of gallic acid [14].

#### 2.4.3. Kinetic Degradation of Phenolic Extracts during Storage Conditions

Among the five polyphenols studied during the stability assay, only rutin and hesperidin could show an isothermal degradation pattern, which often follows first-order kinetics, as shown in Equation (1), where ${\left[Polyphenol\right]}_{o}$ and $\left[Polyphenol\right]$ represent the concentration of the phenolic compound at time 0 and at any time, k refers to the first-order kinetic rate constant, and t stands for time [11].

(1) $$\left[Polyphenol\right]={\left[Polyphenol\right]}_{o}\;e^{\left(-kt\right)}\;$$

Accordingly, the logarithms of both flavonoid concentrations versus time were plotted, yielding linear relationships with an R^2^ between 0.7407 and 0.9456, indicating first-order degradation kinetics. Table 4 reports the estimated rate constants (*k*), the half-life time (t_1/2_), and the correlation coefficients corresponding to the loss of hesperidin and rutin in the lemon and clementine peel extracts during storage for 90 days. As can be seen in Table 4, the *k* values are slightly lower in the extracts preserved at −20 °C. Although it was previously established that there were no significant differences in temperature (other than rutin), it is possible that at 20 °C, these monomeric flavonoids underwent a mild increase by the decomposition of other larger derivatives, whereas this degradation would have been less favored at −20 °C. Accordingly, Li et al. denoted a lower rate of hesperidin degradation when clementine peels were dried at higher temperatures [11]. Inversely, the t_1/2_ parameter, i.e., the time required for the degradation of 50% of the initial compound, increased with higher storage temperatures. This parameter is particularly interesting since it provides compelling data concerning the extract shelf life. Along these lines, it can be safely stated that hesperidin was steadier in the lemon peel extract’s environment (t_1/2,20 °C_ = 71 days) than in that of the clementine’s (t_1/2_,_20°C_ = 45 days), and additionally, rutin decreased its concentration more rapidly than hesperidin did in lemon extracts (t_1/2_,_20°C_ = 62 days), given its lower thermostability [14,30]. At any rate, it is noteworthy that the presence of hesperidin and rutin at 90 days (>42% concentration ratio at 20 °C), as well as the enhanced concentration of coumaric acid, may have contributed to the higher antioxidant power of the lemon extracts compared to that of the clementine ones at 90 days [20].

## 3. Material and Methods

### 3.1. Citrus Samples

*Citrus lemon* L. (lemon) and *Citrus × clementina* (clementine, a hybrid between mandarin and orange), both from the Valencia region (Spain), were acquired from local markets. Firstly, the fruits were cleaned with Milli-Q water, then, the peels were separated by hand and cut into small pieces of a similar size (1 × 0.5 cm) using stainless steel scissors, and finally, they were stored in glass containers at 4 °C until processing.

The initial moisture content of the citrus peels was determined by drying the samples at 105 °C (±0.1 °C) to constant weights [18]. It was determined as a percentage (mean ± standard deviation, *n* = 3) for the lemon (21 ± 1)% and clementine (38.8 ± 0.6)% peels.

### 3.2. Reagents, Solvents and Polyphenol Standards

Analytical-grade reagents and purified water from a Milli-Q system (Merck, Madrid, Spain) were employed in all procedures. Sodium carbonate anhydrous, aluminum chloride 6-hydrate, sodium hydroxide pellets, and sodium nitrite were supplied by Panreac (Barcelona, Spain). Dimethyl sulfoxide (DMSO, ≥99.9%), 2 N Folin-Ciocalteu reagent, and 2,2-diphenyl-1-picrylhydrazyl (DPPH) were obtained from Sigma-Aldrich (St. Louis, MO, USA). Ethanol (EtOH), acetonitrile (ACN), methanol (MeOH), and formic acid (FA) of MS quality were provided by Scharlab (Barcelona, Spain). The phenolic standards, dihydroxy-benzoic acid (≥97.0%), caffeic acid (≥98.0%), *p*-coumaric acid (≥98.0%), *trans*-ferulic acid (98%), gallic acid monohydrate (≥98.0%), kaempferol (≥97.0%), myricetin (≥96.0%), quercetin (≥95.0%), rutin trihydrate (≥95.0%), and resveratrol (≥99.0%) were supplied by Sigma-Aldrich (Madrid, Spain). The analytical standard, hesperidin (≥98.0%) was provided by European Pharmacopoeia. Phenolic stock solutions (200 mg·L^−1^) were prepared in MeOH, 80:20 (*v*/*v*) ethanol-water mixture (for quercetin), or 5% (*v*/*v*) DMSO aqueous solution (for hesperidin). These solutions were stored in the dark at 4 °C or −80 °C for up to two months (of myricetin, hesperidin, *trans*-ferulic, and caffeic acid). Fresh working standard solutions were prepared daily by diluting the stock solutions as required.

### 3.3. Extraction of Polyphenols from Citrus Peel Residues

Phenolic extracts from clementine and lemon peels were obtained according to the method previously described by Gómez-Mejía et al. [12]. Solid–liquid extraction (SLE) was performed on a magnetic stirrer with a ceramic hotplate (model HSC, VELP Scientifica, Usmate, MB, Italy) at 3 rpm and 90 °C for 15 min, using 0.30 g of the citrus peels and 50 mL of the ethanol-water mixture—20:80 (%; v) for the clementine peels and 40:60 (%, v) for the lemon peels. After cooling, the extracts were centrifuged at 10,000 rpm for 10 min (centrifuge 5804, Eppendorf), and then the clear supernatants were collected for the different treatments. Samples were prepared in triplicate.

### 3.4. Storage and Drying Treatments

The effect of the storage temperature on the stability of the phenolic extracts of the lemon and clementine peels was evaluated. For this purpose, 2 mL aliquots of each hydroethanolic extract were taken in amber glass vials equipped with PTFE caps, and kept at −20 °C (frozen preservation) or 20 °C (room temperature preservation) for 90 days. On the other hand, the effect of the drying method on the stability of the phenolic extracts of the citrus waste was also studied at 40 °C and at 60 °C. Thus, 1 mL aliquots of the hydroethanolic extracts were dried by applying two different methods. Oven-drying was carried out using a P-Selecta oven (Panreac) for a maximum of 72 h, while vacuum-drying (Savant SpeedVac, SPD121P-115, Thermo Fisher, Waltham, MA, USA) was carried out for a maximum of 7 h. Finally, the stored and dried extracts (redissolved in 20:80 or 60:40 (%, v) ethanol-water, respectively) were analyzed spectrophotometrically and chromatographically following the procedures described below. All treatments were performed in triplicate.

### 3.5. Determination of Total Polyphenol Content

The Folin–Ciocalteu method was used to assess the total polyphenol content (TPC) of the citrus peel extracts following the treatments [19]. After combining 400 mL of lemon peel extract or 200 mL of clementine peel extract with 70 mL of the Folin–Ciocalteu reagent and 60 mL of 7.5% (*w*/*v*) aqueous Na_2_CO_3_ solution in a final volume of 10 mL, the absorbance of the solution was measured at 720 nm (UV-Vis spectrophotometer Cary 60, Agilent Technologies). External calibration (0–60 μM, *n* = 5) was obtained using gallic acid, as is standard. The results were displayed in terms of mg of gallic acid equivalent per gram of dried peel (mg GAE·g^−1^). This assay was performed in triplicate.

### 3.6. Determination of Total Flavonoid Content

The aluminum trichloride colorimetric assay was used to determine the total flavonoid content (TFC) in the processed extracts [12]. Briefly, 2 mL of water, 150 µL of 5% (w) NaNO_2_, 500 μL of citrus peel extract, and 200 μL of ethanol were mixed and incubated for 5 min. The reagent, AlCl_3_ (150 µL, 10% (w)), was then added to the medium and left for 5 min. Finally, 1 mL of 1 M NaOH was poured to stop the reaction, and after a 15-min-period, the solution was diluted to 10 mL with water. The absorbance of the flavonoid-Al (III) complex was read at 415 nm using a UV-Vis spectrophotometer. Quercetin was employed, as is standard, to obtain the calibration curve (0–45 µM, *n* = 6). The results were presented as milligrams of quercetin equivalent per gram of dry material (mg QE·g^−1^). Samples were examined three times independently.

### 3.7. Antioxidant Activity

The ability of citrus phenolic extracts to scavenge the free radical DPPH was tested as described elsewhere [12]. Several 1-mL working solutions were prepared by combining 500 µL of 0.28 mM DPPH dissolved in methanol, with 100–500 µL of the phenolic extracts and with the appropriate volume of the hydroethanolic mixture, 20:80 (%, v) or 40:60 (%, v), when necessary. The final solutions were stored at room temperature and in the dark for 40 min (lemon extract) or 60 min (clementine extract). After measuring the absorbance at 515 nm, the IC_50_ value was estimated and reported as mg of extract per g of dried sample. Additionally, a DPPH control and a blank control (citrus peel extracts combined with pure MeOH) were also evaluated.

### 3.8. Chromatographic Analysis of Polyphenols

Individual polyphenol determination in the citrus peel extracts was performed by capillary liquid chromatography coupled to a diode array detector and a mass simple quadrupole analyzer (cLC-DAD-MS), following the method previously described by Gómez-Mejía et al. [20] with slight modifications. The extracts were analyzed using an Agilent liquid chromatography system (Mod. 1100 Series) equipped with a G1376A binary capillary pump, a G1379A degasser, a G1315B diode array detector (500 nL, 10 mm pathlength), and a simple quadruple mass spectrometer (6120) equipped with an electrospray ionization source (ESI). An external stainless steel loop (10 μL) was positioned into a Rheodyne^®^ injection valve, and the Agilent Chemstation software package was used for data collection and processing.

The phenolic compounds were separated using a Synergi™ Fusion C18 capillary analytical column (150 × 0.3 mm i.d., 4 μm, Phenomenex, Torrance, CA, USA), a mobile phase consisting of 0.05% (*v*/*v*) formic acid aqueous solution at pH 2.9 (solvent A), and acetonitrile (solvent B), operating in gradient elution mode as follows: 0 to 3 min with 8% of B, 3 to 17 min with 8% to 34% of B, 17 to 21 min with 34% of B, and 21 to 24 min with 8% to 34% to of B. The flow rate was set at 10 μL·min^−1^, and each mobile phase was filtered prior to the analysis with nylon membrane filters (0.22 μm, Teknokroma, Barcelona, Spain).

On-line dual identification was carried out by UV-Vis at five different wavelengths (220, 260, 292, 310, and 365 nm) and by negative ion mode mass detection, selecting [M-H]^−^ as the molecular ion. By contrasting the retention periods, UV-Vis spectra, and molecular ions obtained from the standards, the phenolic compounds present in the sample extracts were identified. External calibration curves were obtained for quantitative analyses by DAD or MS according to the highest sensitivity and/or selectivity. Furthermore, to attain analyte on-column focusing, injection solutions were prepared by diluting 50–200 μL of sample aliquots, 50 μL of acetonitrile, and 800 μL of methanol with a 0.05% (*v*/*v*) formic acid aqueous solution at pH 2.9. Sample extracts were analyzed in triplicate.

The performance of the method was evaluated for the standard solutions under optimal chromatographic conditions in terms of linear range, limits of detection (LOD) and quantification (LOQ), and precision [21,22]. Linear ranges (*n* ≥ 5) were set at concentrations between 0.5 and 450 μg·L^−1^, chromatographic peak areas were analyzed by linear least-squares regression, and linearity was assessed as R^2^ values. LOD and LOQ were calculated for analyte concentration at the height as 3 signal-to-noise (*S/N*) and 10 *S/N*, respectively. The precision was estimated as 30 μg·L^−1^ for gallic acid, dihydroxybenzoic acid, *trans*-ferulic acid, and resveratrol, 20 μg·L^−1^ for caffeic acid, 50 μg·L^−1^ for *p*-coumaric acid, 110 μg·L^−1^ for rutin, 65 μg·L^−1^ for hesperidin, myricetin, and kaempferol, and 70 μg·L^−1^ for quercetin. Intra-day variation (*n* = 3) was assessed by injecting three standard solutions at the target concentration for each analyte on the same day. Inter-day precision was similarly obtained from three successive days (N = 9, three injections per day). The relative standard deviation (RSD, %) was taken as a measure of repeatability, and intermediate precision was calculated for both the retention factor (*k*) and the peak areas of each analyte.

### 3.9. Statistical Analysis

Data were statistically analyzed by a two-tailed paired t-Student test, a multi-factorial analysis of variance (ANOVA), and a principal component analysis (PCA) using the software package Statgraphics 19 (Statgraphics Technologies. Inc., Rockville, MD, USA).

## 4. Conclusions

The results presented in this study provide useful information for the preservation and storage of polyphenolic extracts from citrus peels, which could be exploited for industrial application. The drying method significantly affected the phenolic content and the antioxidant activity of the clementine and lemon peel extracts. Hence, vacuum-drying at 60 °C for 6 h was suitable for the preservation of hesperidin, *p*-coumaric, and *trans*-ferulic acids, as well as for improving the antioxidant activity of the clementine peel extracts, while oven-drying at 40 °C guaranteed higher recoveries of TPC, hesperidin, and rutin in the lemon peel extracts. As for storage conditions, the significant fluctuation in the phenolic profiles observed in both of the liquid extracts were independent of temperature, except for gallic acid and rutin. Lower values of *k* were estimated for the degradation of hesperidin and rutin at 20 °C, indicating that these extracts can be stored unfrozen. In general, these extracts can be stored for up to 51 days without losing more than 50% of the initial concentration. Therefore, the drying method and temperature should be carefully selected according to the bioactive compounds expected to be obtained after the processing and/or stocking of the liquid phenolic extracts.

## Figures and Tables

**Table 1:** cLC-DAD-MS calibration curves, spectral data, and wavelengths for the identification and quantification of polyphenols.

Compound	Retention Time, min	Wavelength (λ_max_), nm	Molecular Ion [M-H]ˉ (*m*/*z*) (Fragmentor, eV)	Calibration Equation, *y* = *a* · *x* + *b*	
				*a*, μg·L^−1^	*b*
Gallic acid	3.6	260	169 (70)	(4.4 ± 0.4) × 10^2^	(0 ± 8) × 10^3^
DHB ^1^	4.9	260	153 (70)	(5.4 ± 0.4) × 10^2^	(0 ± 7) × 10^3^
Caffeic acid	11.8	310	179 (150)	(6 ± 1) × 10^2^	(0 ± 6) × 10^3^
*p*-Coumaric acid	14.5	310	163 (70)	(1.06 ± 0.06) × 10^3^	(0 ± 3) × 10^3^
*trans*-Ferulic acid	15.4	310	193 (70)	(4.1 ± 0.5) × 10^2^	(0 ± 2) × 10^3^
Rutin	15.0	260	-	0.8 ± 0.1	(1.8 ± 0.7) × 10^3^
Hesperidin	16.0	292	-	0.85 ± 0.03	(2 ± 2) × 10
Myricetin	17.7	365	317 (150)	0.7 ± 0.1	(0 ± 1) × 10
Resveratrol	18.5	310	227 (150)	5.0 ± 0.4	(4 ± 2) × 10
Quercetin	19.7	365	301 (150)	(6.2 ± 0.6) × 10^2^	(0 ± 4) × 10^3^
Kaempferol	22.7	365	284 (150)	(1.21 ± 0.07) × 10^3^	(6 ± 4) × 10^3^

^1^ DHB: Dihydroxybenzoic acid.

**Table 2:** cLC-DAD-MS calibration curves, spectral data, and wavelengths for the identification and quantification of polyphenols.

Compound	Linear Range (*n*), µg∙L^−1^	R^2^	LOD, µg∙L^−1^	LOQ, µg∙L^−1^	Intra-Day Repeatability (*n* = 3) RSD (%)		Inter-Day Repeatability (N = 9) RSD (%)	
					*k*	Area	*k*	Area
Gallic acid	20–450 (8)	0.9937	4	13	4.8	3.5	5.1	4.5
DHB ^1^	21–450 (9)	0.9951	6	20	3.8	5.3	6.1	5.8
Caffeic acid	16–70 (5)	0.9900	4	13	2.4	3.9	6.8	4.1
*p*-Coumaric acid	5–180 (9)	0.9931	0.7	2	0.77	4.0	1.0	5.9
*trans*-Ferulic acid	16–60 (5)	0.9948	4	13	0.41	3.9	0.70	4.5
Rutin	70–160 (5)	0.9986	20	67	0.40	3.3	1.1	3.6
Hesperidin	50–160 (7)	0.9992	8	27	0.48	1.0	1.0	1.0
Myricetin	70–120 (5)	0.9978	20	67	0.32	4.4	0.60	5.2
Resveratrol	16–60 (5)	0.9938	4	13	0.34	3.5	0.60	3.9
Quercetin	1–150 (8)	0.9916	0.1	0.3	0.32	4.5	0.59	5.2
Kaempferol	0.5–150 (9)	0.9958	0.1	0.3	0.54	4.1	1.1	5.0

^1^ DHB: Dihydroxybenzoic acid.

**Table 3:** Phenolic compounds and estimated values of total content and antioxidant activity of raw extracts obtained from lemon and clementine peels. Data are expressed as mean ± standard deviation (*n* ≥ 6).

Compound (µg·g^−1^ Sample)	Lemon Peels	Clementine Peels
Gallic acid	68 ± 15	98 ± 13 **
DHB ^1^	*n.q.*	*n.d.*
Caffeic acid	*n.q.*	*n.d.*
*p*-Coumaric acid	178 ± 23	258 ± 90 **
*trans*-Ferulic acid	177 ± 15	438 ± 125 **
Rutin	(1.33 ± 0.06) × 10^3^	*n.d.*
Hesperidin	(8.7 ± 0.9) × 10^3^	(1.5 ± 0.5) × 10^4^ **
Myricetin	*n.q.*	*n.d.*
Resveratrol	*n.q.*	*n.d.*
Quercetin	*n.q.*	*n.d.*
Kaempferol	5 ± 1	2.70 ± 0.02 **
Total Phenolic Acids (mg·g^−1^ sample)	0.42 ± 0.01	0.6 ± 0.1 **
Total Flavonoids (mg·g^−1^ sample)	10.0 ± 0.8	15 ± 5 **
Total Polyphenols (mg·g^−1^ sample)	10.4 ± 0.8	16 ± 5 **
TPC (mg GAE·g^−1^ sample DW)	2.1 ± 0.4	6.2 ± 0.8 **
TFC (mg QE·g^−1^ sample DW)	30 ± 3	40 ± 2 **
DPPH (IC_50_, mg·g^−1^ sample DW)	0.61 ± 0.08	0.95 ± 0.03 **

^1^ DHB: Dihydroxybenzoic acid; *n.q.*: determined at the levels of the method detection limit (not quantifiable); *n.d.*: non-detected; GAE: gallic acid equivalents; QE: quercetin equivalents; DW: dry weight. ** Statistical differences < 0.05 by applying a two-tailed paired *t*-Student test.

**Table 4:** Kinetic parameters of hesperidin and rutin degradation during storage of lemon and clementine peel extracts over 90 days.

Peel Extract	Polyphenol	Temperature, °C	*k*, Day^−1^	t_1/2_, Day	R^2^
Lemon	Hesperidin	−20	0.0122	57	0.9456
		20	0.0098	71	0.8773
	Rutin	−20	0.0133	52	0.8963
		20	0.0112	62	0.7407
Clementine	Hesperidin	−20	0.0205	34	0.9216
		20	0.0154	45	0.7301

## Data Availability

The data presented in this study are available on request from the corresponding author.

## Supplementary Materials

The following supporting information can be downloaded at: https://www.mdpi.com/article/10.3390/molecules28041624/s1, Table S1: Values of TPC and TFC parameters found in the hydroethanolic extracts of citrus peels during different drying treatments. Table S2: Values of TPC, TFC and DPPH parameters determined in hydroethanolic extracts of citrus peels preserved at room temperature (control) and frozen.

## Sample Availability

Samples of the compounds are not available from the authors.
