# Supplementary material for: Effect of Storage and Drying Treatments on Antioxidant Activity and Phenolic Composition of Lemon and Clementine Peel Extracts
Source: Molecules. 2023 Feb 8;28(4):1624. doi: 10.3390/molecules28041624 (PMC9958772; doi:10.3390/molecules28041624)
Supplement: Supplementary file 1 [file molecules-28-01624-s001.zip › Suplementary material.pdf]

Table S1. Values of TPC and TFC parameters found in the hydroethanolic extracts of citrus peels during different drying treatments.

Drying conditions	Lemon peel extract			Clementine peel extract		
	Time (h)	TPC (mg GAE·g^{-1})	TFC (mg QE·g^{-1})	Time (h)	TPC (mg GAE·g^{-1})	TFC (mg QE·g^{-1})
**Raw extract**	0	2.1 ± 0.4	30 ± 3	0	6.2 ± 0.8	40 ± 2
**Oven drying at 40 °C**						
	2	2.6 ± 0.4	28 ± 2	2	9 ± 1	43 ± 3
	4	1.1 ± 0.1	28 ± 2	4	4.6 ± 0.2	32 ± 3
	6	2.1 ± 0.2	30 ± 1	6	5.0 ± 0.4	41 ± 4
	24	1.0 ± 0.2	27 ± 1	24	10.6 ± 0.4	34 ± 1
	48	1.8 ± 0.3	27 ± 1	48	8.1 ± 0.1	34 ± 2
	72	2.8 ± 0.3	25 ± 1	72	6.0 ± 0.4	34 ± 3
**Oven drying at 60 °C**						
	2	2.1 ± 0.2	30 ± 3	2	8 ± 1	39 ± 4
	4	2.4 ± 0.3	23 ± 1	4	1.9 ± 0.4	35 ± 2
	6	1.8 ± 0.3	29.2 ± 0.5	6	2.9 ± 0.3	33 ± 3
	24	2.2 ± 0.2	27 ± 3	29	6 ± 1	34 ± 2
**Vacuum-drying at 40 °C**						
	2	2.6 ± 0.4	28.5 ± 0.7	2	12.4 ± 0.5	40 ± 3
	4	1.1 ± 0.1	29 ± 1	4	8 ± 1	37 ± 3
	6	2.1 ± 0.2	30 ± 1	7	5 ± 1	39 ± 3
**Vacuum-drying at 60 °C**						
	1	1.7 ± 0.1	28 ± 2	2	5 ± 1	38 ±1
	2	1.8 ± 0.2	34 ± 1	4	4 ± 1	37 ± 3
	4	2.1 ± 0.2	33 ± 2	6	5.9 ± 0.5	36 ± 2

Table S2. Values of TPC, TFC and DPPH parameters determined in hydroethanolic extracts of citrus peels preserved at room temperature (control) and frozen.

		**Lemon peel extract**			**Clementine peel extract**		
**Storage conditions**	**Time (days)**	**TPC (mg GAE·g^{-1})**	**TFC (mg QE·g^{-1})**	**DPPH (mg·g^{-1})**	**TPC (mg GAE·g^{-1})**	**TFC (mg QE·g^{-1})**	**DPPH (mg·g^{-1})**
**Raw extract**	0	2.1 ± 0.4	30 ± 3	0.61 ± 0.08	6.2 ± 0.8	40 ± 2	0.95 ± 0.03
**Control extract (20 °C)**							
	3	2.7 ± 0.1	34.2 ± 0.3	0.61 ± 0.01	4.9 ± 0.1	33.1 ± 0.4	1.1 ± 0.1
	7	2.5 ± 0.2	36.3 ± 0.9	0.95 ± 0.03	3.0 ± 0.1	28 ± 2	1.6 ± 0.1
	15	2.0 ± 0.1	38 ± 1	0.72 ± 0.02	3.6 ± 0.2	33 ± 3	1.25 ± 0.03
	28	2.0 ± 0.2	36.3 ± 0.8	0.67 ± 0.01	2.5 ± 0.1	37 ± 2	1.05 ± 0.08
	51	2.3 ± 0.2	33 ± 2	0.38 ± 0.01	3.2 ± 0.1	35 ± 1	1.1 ± 0.1
	65	2.5 ± 0.4	35.8 ± 0.9	0.61 ± 0.02	3.7 ± 0.2	34 ± 1	1.27 ± 0.03
	90	2.6 ± 0.3	38 ± 1	0.60 ± 0.01	3.5 ± 0.2	31 ± 1	1.47 ± 0.07
**Frozen extract (- 20 °C)**							
	3	2.8 ± 0.1	33.3 ± 0.6	0.75 ± 0.01	5.0 ± 0.1	32.6 ± 0.5	1.1 ± 0.1
	7	2.9 ± 0.1	34 ± 1	0.52 ± 0.02	4.7 ± 0.1	28 ± 2	1.81 ± 0.01
	15	2.5 ± 0.1	34 ± 1	0.59 ± 0.04	4.8 ± 0.2	32 ± 2	1.21 ± 0.04
	28	2.5 ± 0.2	32.1 ± 0.5	0.57 ± 0.01	5.3 ± 0.2	36 ± 2	1.2 ± 0.1
	51	1.9 ± 0.3	31 ± 1	0.23 ± 0.02	5.8 ± 0.1	35 ± 1	1.10 ± 0.08
	65	2.3 ± 0.1	34 ± 3	0.43 ± 0.01	6.1 ± 0.3	34 ± 1	0.76 ± 0.04
	90	3.1 ± 0.3	37 ± 2	0.62 ± 0.05	5.9 ± 0.4	36 ± 1	1.32 ± 0.04
